# Determinants of adherence to personal preventive behaviours based on the health belief model: a cross-sectional study in South Korea during the initial stage of the COVID-19 pandemic

**DOI:** 10.1186/s12889-022-13355-x

**Published:** 2022-05-11

**Authors:** Jiwon Baek, Kyung Hee Kim, Jae Wook Choi

**Affiliations:** 1 Institute for Environmental Health, Korea University, 73, Inchon-ro, Seongbuk-gu, Seoul, 02841 South Korea; 2 Department of Preventive Medicine, Korea University College of Medicine, 73, Inchon-ro, Seongbuk-gu, Seoul, 02841 South Korea

**Keywords:** Preventive behaviours, Health belief model, Cues to take action, COVID-19, South Korea

## Abstract

**Background:**

Along with the rapid transmission of COVID-19, adherence to preventive behaviours plays a crucial role with respect to the control of COVID-19. However, different individuals’ psychological characteristics and risk perception result in various forms of response to preventive behaviours. Based on the Health Belief Model, this study identifies the factors associated with preventive behaviours towards COVID-19 in South Korea during the initial stage of the COVID-19 pandemic.

**Methods:**

A cross-sectional study was conducted in April 2020 through an anonymous online survey. A total of 1207 people in the age bracket of 20–59 years participated in the survey. Single and multiple linear regression analyses were conducted to identify the determinants of preventive behaviours against COVID-19.

**Results:**

The following factors were associated with preventive behaviours towards COVID-19: female gender (β = .124, *p* < 0.001), has a master’s degree or above (β = 0.065, *p* = 0.010), perceived susceptibility (β = .197, *p* < 0.001), self-efficacy (β = .404, *p* < 0.001), trust in radio (β = −.080, *p* = .006), trust in official government website (β = .057, *p* = .045), trust in social networks (β = .054, *p* = .033), and trust in family and friends (β = .068, *p* = .009), with an explanatory power of 41.5% (R^2^ = 0.415).

**Conclusions:**

To flatten the epidemic curve, it is important to understand the public’s risk perception and the motivation behind behavioural responses that aim to promote preventive behaviours among the public. Thus, this study calls for the provision of accessible and credible information sources and demonstrates a public health campaign that encourages the public’s engagement in preventive behaviours towards COVID-19.

**Supplementary Information:**

The online version contains supplementary material available at 10.1186/s12889-022-13355-x.

## Background

Since the outbreak of coronavirus disease in Wuhan, China, in December 2019 [1], the numbers of confirmed COVID-19 cases and deaths have rapidly surged**.** In South Korea, the first confirmed cases occurred on 4 January 2020 [2], and the World Health Organization declared the insurgence of COVID-19 a global health emergency on 31 January 2020. Along with the rapid transmission of COVID-19, there is the issue of the large number of people suffering from COVID-19 [3]. In the absence of treatment or vaccination in 2020, adherence to preventive behaviours was reported to have been helpful in flattening the epidemic curve [4, 5]. Beginning in March 2020, the WHO announced a number of infection prevention guidelines, such as those on hand washing, physical distancing, and wearing a mask. Even after the COVID-19 pandemic is over, there is a possibility that other diseases will arise and become prevalent before any remedies for them are suggested. From this point of view, it is significant to study how people engage in preventive behaviours and to make people adopt such behaviours during the initial stage of a disease.

To be able to control the spread of COVID-19, health authorities have urged the public to abide by preventive behaviours. Reflecting on the past, people’s responses to pandemic regulations have been presented in various formats. For example, when the 2003 SARS outbreak took place in Hong Kong, approximately 75% of the people followed personal preventive behaviours, such as wearing masks and washing hands [6]. In contrast, when the 2009 influenza A/H1N1 epidemic occurred, only 21.5% of the Hong Kong survey participants reporting wearing a face mask when they had an influenza-like disease in the early stage of the epidemic [7]. Moreover, in response to the COVID-19 pandemic, 81.8% of American and Australian participants were reported to have complied with the stay-home regulations [8].

According to previous studies, different individuals’ psychological characteristics and risk perceptions result in various forms of responses to preventive behaviours. For example, Shahnazi et al. found that psychological responses such as perceived benefits and self-efficacy positively influence the adoption of preventive behaviours [9]. Additionally, De Zwart et al. demonstrated that perceived levels of higher risk are related to engagement in more preventive behaviours [10]. Although many researchers have reached the agreement that psychological responses and risk perceptions influence individual preventive behaviour, most of their studies have not examined how the psychological characteristics and perception of risk can be affected by different knowledge and information sources. Given the importance of understanding the public’s perception of health risks and of offering reliable information sources to the public, this study aims to explore the role of Health Belief Model (HBM) factors in facilitating preventive health behaviours towards COVID-19 among South Koreans [11–14].

The HBM model was originally developed to study why patients do not adopt preventive behaviours such as medication and vaccination to minimize their health threat [15]. The Health Belief Model is used as a conceptual framework to explain how psychological characteristics, risk perception and cues are related to adherence to preventive behaviours. Therefore, considering the HBM components, which consist of perceived susceptibility, severity, benefits, barriers, self-efficacy, and cues to take action, helps us to understand health behaviours and to suggest suitable interventions to encourage people adhere to preventive behaviours [16]. Since the HBM is the most representative model and many other models have been based on its structure, if we find that the HBM model is useful when applied to relevant situations, then our modelling work can easily be expanded to other models in follow-up works. Such expansion would be especially helpful when people are faced with new situations, such as the SARS and H1N1 pandemics, and researchers are able to analyse their changes in health behaviour using the HBM model [1, 4, 9].

For this study, we scrutinize the relationship between reliable information sources and preventive behaviours from the sociocultural perspective. Our study results are expected to provide appropriate communication strategies to overcome the infectious COVID-19 epidemic and offer a basis for policy recommendations.

## Methods

### Data source

We administered a cross-sectional survey. To evaluate the public’s health behaviour responses to COVID-19, we conducted an anonymous online survey. To increase the representativeness of the sample, a proportional stratified sampling that reflected age, gender, and population region in the sample quota ratios was used. The number of participants was set based on the composition of the registered resident population announced by Statistics South Korea in February 2020. A total of 1406 patients visited the online survey between April 14 and April 20, 2020.

In South Korea, the first confirmed COVID-19 cases occurred on 4 January 2020, which prompted the Korea Disease Control and Prevention Agency to implement the ‘New Normal Level’ and strengthen their surveillance [17]. When the fourth confirmed case occurred on 28 January 2020, the KCDC scaled up the alert level and conducted publicity campaigns about taking preventive behaviours against infectious disease. During the month of February, the number of confirmed cases increased radically as the new infectious disease rapidly spread nationwide, even at the local community level. During this period, the health authorities conducted various campaigns on personal preventive behaviours through posters, digital images, and text messages. In particular, they suggested specific health behaviours according to place and time in order to induce people to adhere to health behaviours**.**

To evaluate the public’s health behaviour responses to COVID-19, we developed a questionnaire made up of 26 questions (5 sociodemographic questions; 5 health behaviour questions; 9 Health Belief Model questions and 7 questions about cues to take action), and we conducted online research using a research panel. An online research panel is a sample of persons who have already agreed to take surveys on websites. Since these individuals have already agreed to provide their sociodemographic information, it is easy for researchers to maintain an appropriate balance across age, gender, and population region. Additionally, based on a privacy policy, specific personal information about the respondents such as their name and address are not permitted to be exposed to the researchers.

The data for the analysis were collected by an online research company named “TRUIS”, which maintains 420,000 online panels [18]. According to the composition of the registration population announced by the National Statistics Office in February 2020, we set gender, age group, and regional quota ratios. Before beginning the survey, TRUIS set gender, age group, and regional quota ratios based on registration population data for impartial analysis. At first, 1406 people had accessed the online survey. A total of 102 participants exceeded the quota ratio, so we excluded them from the survey. For example, assuming that 1406 subjects were subject to the survey, 165 people were allocated to the quota when applying the ratio of the number of men in their 20s. Thus, if 200 men in their 20s answered the questions, then the results of the survey could be biased. This means that, in this example, 35 respondents would need to be excluded from the sample. Ultimately, a total of 1304 respondents completed the questionnaire; however, among them, 66 respondents did not complete the survey, and 31 respondents did not provide consistent responses. Therefore, we concluded that 1207 respondents were credible based on the quota ratios, which resulted in a response rate of 92.5%.

The final sample size was 1207, with a considerable margin error of 2.82% and a 95% confidence interval. Since this study analysed people’s autonomous actions or responses to COVID-19, which means that they needed to be able to decide their actions on their own, it was important to choose adults as the respondents. For this reason, the survey decided to provide comprehensive information about the adult population in the age bracket of 20–59 years. Prior to the survey, participants agreed to the provision that the contents and purpose of this study were understood and that they were willing to participate in the study. Anonymous participation was strongly mandated, and no identifiable information was collected from the respondents.

### Outcome measure

To evaluate the degree of the respondents’ adherence to COVID-19 preventive behaviours, we analysed their responses to the personal preventive measures recommended by the World Health Organization (WHO). The WHO developed a comprehensive strategy to control COVID-19 that is made up of a list of actions recommended for individuals, communities, governments, and international bodies to suppress the spread of the SARS-CoV-2 virus [19]. Of these actions, we focused on the individual aspects of the preventive measures to assess the respondents’ beliefs and perceptions concerning preventive behaviours regarding COVID-19. Consequently, we used five items, namely, frequent hand hygiene, respiratory etiquette, wearing a mask, environmental cleaning at home, and self-quarantine. The answers were rated on a 7-point scale ranging from 1 = strongly disagree to 7 = strongly agree. The total value of the precautionary behaviours was calculated by averaging the scores of each of the questions. To measure internal consistency, a reliability analysis was carried out on the preventive behaviours scale comprising 5 items. The Cronbach’s alpha value for the survey was .75, which indicated an acceptable level of reliability.

### Independent variables

By building upon the HBM from previous literature, we developed a total of eight categories of determinants that influenced the preventive behaviours taken towards preventing COVID-19. The structured variables covered sociodemographic information, perceived susceptibility, perceived severity, perceived benefit, perceived barrier, self-efficacy of preventive behaviours, and cues to take action. In particular, the sociodemographic characteristics of the survey participants included gender, age, education level, monthly household income, and marital status.

The second part of this study was based on the HBM. The study participants were asked to provide their opinions on specific statements. Perceived susceptibility, severity, benefits, and barriers were each evaluated. To measure the HBM factors, except for self-efficacy, the respondents are asked to answer the two separate questions. The final scores from each factor were obtained by averaging each score. If the final score was above the average score, this was considered indicative of each factor being at a high level. Perceived susceptibility refers to one’s belief regarding the possibility of being infected (e.g., If I do not take precautions, I think I will be more likely to be infected with COVID-19). Perceived severity refers to one’s belief in the seriousness of the infection (e.g., If I am infected with the SARS-CoV-2 virus, it will impact me severely) [20]. Perceived benefits refer to the efficacy of preventive behaviours in reducing the risk of being infected by the SARS-CoV-2 virus (e.g., If I follow the preventive behaviours, doing so will reduce the risk of getting infected with COVID-19). In contrast, perceived barriers represent the obstacles that inhibit the implementation of preventive behaviours (e.g., It is annoying and uncomfortable to follow preventive behaviours) [21]. The answers were scored on a scale ranging from 1 to 7 (1 = strongly disagree, 7 = strongly agree).

Self-efficacy refers to an individual’s confidence in successfully carrying out preventive health behaviours for the prevention of COVID-19 (e.g., I am able to follow the preventive behaviours) [22]. The survey participants were asked to assess their self-efficacy through a question, and they were asked to indicate their level of agreement using a 7-point Likert scale.

Finally, the HBM assumes that people are set in motion through cues to take action. These cues to take action trigger individuals to take action by using various sources [23]. We chose seven items to evaluate the survey participants’ trust cues that could affect their preventive behaviours. The respondents were asked to indicate how much they trusted the following sources of information with regard to the information provided about COVID-19: printed media, radio, television, health care providers, official government website, social networks, and family and friends. The answers were scored from 1 (do not trust at all) to 7 (trust completely). The scores were obtained by averaging the scores of the seven questions. To measure internal consistency reliability, we calculated the Cronbach’s alpha coefficient on items of the Health Belief Model and cues to take action. The Health Belief Model subscale consisted of 9 items and α = .71, while the cues to take action subscale consisted of 7 items and α = .79. Each of the Cronbach’s alpha values showed that the questions reached acceptable levels of reliability.

### Statistical analyses

A descriptive analysis was conducted to illustrate the general characteristics of the study sample using the frequencies and percentages of the categorical variables. We conducted single and multiple linear regression analyses to identify the factors that affected the respondents’ health behaviours towards COVID-19 prevention. The data were analysed using IBM SPSS software version 22.

## Results

### Background characteristics of the study population

Table 1 summarizes the sociodemographic characteristics of the survey respondents. In total, there were 616 men (51%) and 591 women (49%). The mean age of the respondents was 40.1 years (M = 40.1, SD = 11.06). A majority of the respondents had a bachelor’s degree or above (75.5%), and 67% of them had a monthly income of over KRW 3,000,000. Approximately half (52%) of the respondents reported being married.

**Table 1:** Sociodemographic characteristics of the survey respondents (*n* = 1207)

Variables	N	%
Gender		
Male	616	51
Female	591	49
Age groups in years		
20–29	275	22.8
30–39	278	23
40–49	329	27.3
50–59	325	26.9
Education		
Less than a bachelor’s degree	296	24.5
Bachelor’s degree	461	38.2
Master’s or higher	450	37.3
Monthly household income (10,000 KRW)^a^		
< 300	397	32.9
300 < 600	549	45.5
≥ 600	261	21.5
Marital status		
Married	628	52
Never married	537	44.5
Divorced/widowed/separated	42	3.5

^a^*KRW* Currency of Korea (1000 KRW is approximately 1 USD)

### Personal preventive behaviours

**Fig. 1:**
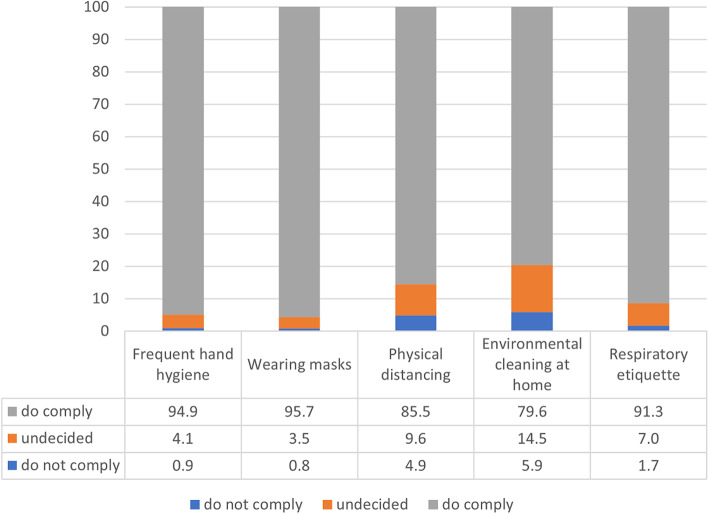
Frequencies of preventive behaviour

Figure 1 describes the results of the frequency distribution analysis of the personal preventive behaviours recommended by the WHO. These items, which were measured using a seven-point scale, were divided into three groups: Group 1 (“do not comply”) includes the respondents who felt uncomfortable complying with the preventive behaviours (1 to 3 points); Group 2 (“neutral”) includes all the respondents who replied that they were “undecided” (4 points); and Group 3 (“do comply”) includes the respondents who agreed to comply with the preventive behaviours (5 to 7 points).

The survey found that more than 89.4% of the respondents engaged in personal preventive behaviours. Most of the respondents wore masks (95.7%), frequently engaged in hand washing (94.9%), and covered their mouths while coughing (91.3%).

### Factors associated with personal preventive behaviours

We conducted a simple regression analysis by separately setting the sociodemographic characteristics, the Health Belief model, and the cues to take action factors as the explanatory variables. The dependent variable was a simple calculated mean of the personal preventive behaviours, which are shown in Fig. 1, including respiratory etiquette, environmental cleaning at home, physical distancing, wearing masks and frequent hand hygiene. Table 2 presents the results of the simple linear regression analysis. The univariate analysis indicated that all the independent variables, except monthly household income, were significantly associated with personal preventive behaviours taken to prevent COVID-19.

**Table 2:** Results of the simple linear regression analysis

Independent variables		*β*	*P* value
	**Gender (female)**	1.634	<.001
	**Age group in years**		
	20–29	−.732	.021
	30–39	−.398	.206
	40–49	−.207	.493
	50–59	(Reference)	
	**Education level**		
	Less than a bachelor’s degree	.238	.407
**Sociodemographic characteristics**	Bachelor’s degree	(Reference)	
	Master’s or higher	.756	.003
	**Monthly household income**		
	< 300	−.037	.885
	300 < 600	(Reference)	
	≥ 600	.090	.756
	**Marital status**		
	Never married	−.785	.001
	Married	(Reference)	
	Divorced/widowed/separated	−.342	.576
**Health belief model**	**Perceived susceptibility**	1.533	<.001
	**Perceived severity**	1.086	<.001
	**Perceived benefits**	1.526	<.001
	**Perceived barriers**	−.233	.002
	**Self-efficacy**	2.113	<.001
**Cues to take action**	**Print media**	.343	<.001
	**Radio**	.249	.003
	**Television**	.702	<.001
	**Health care providers**	.733	<.001
	**Official government website**	.734	<.001
	**Social networks**	.176	.020
	**Family and friends**	.662	<.001

The significant variables from the simple linear regression analysis were included in the multiple linear regression analysis to identify the factors associated with personal preventive behaviours. These variables accounted for approximately 41.5% of the variance found in the preventive behaviours.

As shown in Table 3, the eight factors that had statistically significant effects on COVID-19 preventive behaviours were as follows: female gender (β = .124, *p* < 0.001); holding at least a master’s degree or above (β = 0.065, *p* = 0.010); perceived susceptibility (β = .197, *p* < 0.001); self-efficacy (β = .404, *p* < 0.001); trust in the radio (β = −.080, *p* = .006); social networks (β = .054, *p* = .033); official government website (β = 0.057, *p* = .045); and family and friends (β = .068, *p* = .009). However, other factors, such as age, income, perceived severity, perceived benefits, perceived barriers, trust in print media, trust in television, and trust in health care providers were not associated with preventive behaviours.

**Table 3:** Results of the multivariate linear regression analysis of preventive behaviours

	Variables		*B*	SE	*β*	*P* value
Sociodemographic factors	Gender	Female	.960	.177	.124	<.001
	Age in years	20–29	−.054	.312	−.006	.862
		30–39	.283	.267	.031	.289
		40–49	.277	.241	.032	.252
		50–59	(Reference)			
	Education	Less than a bachelor’s degree	.235	.229	.026	.306
		Bachelor’s degree	(Reference)			
		Master’s or above	.519	.200	.065	.010
	Marital status	Married	(Reference)			
		Never married	−.023	.224	−.003	.919
		Divorced/widowed/separated	−.392	.485	−.019	.420
Health belief model factors	Perceived susceptibility		.655	.099	.197	<.001
	Perceived severity		.172	.092	.049	.062
	Perceived benefits		.103	.109	.029	.342
	Perceived barriers		−.094	.061	−.036	.125
	Self-efficacy		1.504	.105	.404	<.001
Cues to take action	Reliable channels	Print media	.075	.085	.026	.375
		Radio	−.232	.084	−.080	.006
		Television	.104	.098	.032	.289
		Social networks	.142	.067	.054	.033
	Reliable resources	Health care providers	−.020	.097	−.006	.838
		Official government website	.172	.086	.057	.045
		Family and friends	.238	.091	.068	.009

## Discussion

Our study revealed the relationships that exist between HBM factors and COVID-19 preventive behaviours in South Korea. The results of this study show that perceived susceptibility and self-efficacy positively influence the adoption of preventive behaviours. Therefore, it is important for the government and health officials to provide reliable information about pandemic status and guidelines for adherence to preventive behaviours. In addition, our study found that trust information channels and trust information sources have effects on preventive behaviours. Understanding the characteristics of those who trust a particular information channel or source might help persuade these individuals to engage in preventive behaviours. Our results also identified that sociodemographic factors such as age and education level influence preventive behaviours. Given the severe threats presented by COVID-19, performing preventive behaviours in the early stage of the pandemic played a key role in slowing the spread of the disease.

First, our study analysed personal preventive behaviours. Most of our study respondents engaged in preventive behaviours. Previous Asian epidemic outbreaks and Asian cultural characteristics may have influenced the respondents’ active engagement in preventive behaviours. Since a large number of Asian countries have already experienced numerous other infectious disease outbreaks, many Asians hold the belief that intervention protects them from viral infection [24]. As per the study of Tong (2020), East Asians tend to be more engaged in personal preventive behaviours because of Asian health beliefs [25].

Second, we examined the factors associated with personal preventive behaviours. Factors such as gender, education level, perceived susceptibility, self-efficacy, trust in information from the radio, trust in information from social networks, trust in the official government website, and trust in family and friends were significant predictors of preventive behaviours. This study identified that belief factors and credible information sources are related to preventive behaviours and highlighted the importance of providing an appropriate communication strategy to the public to help them remain engaged in adherence to preventive behaviours.

Our findings have shown how sociodemographic factors influence the adoption of preventive behaviours. In our analysis, being a woman was found to be a significant factor affecting preventive behaviours. Since women are more likely to be health-conscious and risk-averse, women tend to better comply with preventive behaviours towards infectious diseases than do men [6, 26]. For example, women’s adherence to preventive behaviours was found to be stronger than that of men in Hong Kong [7], Serbia [27], and South Korea [28] during the COVID-19 pandemic. Furthermore, individuals with higher educational backgrounds tend to abide by preventive behaviours. This may be caused by the fact that individuals with higher education levels are more likely to have a better assessment of risk management and are more likely to be exposed to information relating to preventive behaviours [14]. A study by Leung found that survey participants with higher education levels tended to abide by preventive behaviours in the case of the 2003 SARS outbreak in Hong Kong [6]. Another possible explanation for this outcome is that a longer education period tends to provide better training to adhere to government-recommended behaviours [29].

Of all the HBM factors, our study found perceived susceptibility and self-efficacy to be important determinants in adherence to preventive behaviours. Perceived susceptibility refers to an individual’s evaluation of health risks, while risk perception can influence the level of an individual’s preventive behaviours [30–32]. However, this differs from the pattern seen in previous studies where perceived severity is strongly associated with compliance with preventive actions. This may be due to the characteristics of COVID-19. While COVID-19 is highly contagious, it does have a relatively low mortality rate. This is perhaps the reason people are worried about being infected by SARS-CoV-2. However, they regard it as a challenge that society has to overcome. Another possible explanation is the difference in the data collection timing. In the early phase of the epidemic, people panicked because of the limited amount of reliable information. As time has passed, people have come to be exposed to much information, and they have come to realize the pandemic’s status and the importance of preventive behaviours. This is why it is important for the government and health officials to share and communicate credible information about the pandemic status with the public. In our study, self-efficacy was found to be the greatest factor in explaining compliance with preventive behaviours. According to the HBM, self-efficacy is a strong predictor of health-promoting behaviours [33, 34]. Self-efficacy refers to an individual’s ability to successfully carry out behaviour change [22]. This result is in alignment with the extant literature that suggests that the communication strategy of pandemics should increase an individual’s confidence in their capacity to adopt preventive behaviours [14].

Trust in different information channels influences the levels of knowledge and beliefs regarding the COVID-19 pandemic [35]. Our study has shown that trust in information from the radio has a negative effect on preventive behaviours. As per the study by Hwang on the patterns among South Korean radio listeners, the number of radio listeners is gradually decreasing, and fewer listeners use the radio to acquire knowledge and information [36]. Given the passivity of the radio communication type, guiding people’s health behaviour via the use of radio information is more difficult than in other forms of media [37]. Another possible explanation could be related to the listeners’ political stance. The majority of radio listeners in South Korea listen while they are in their vehicles [36]. This implies that most of the radio listeners own their own vehicles. The minimum required driving age in South Korea is 19, which is relatively higher than that in Western countries. In addition, because of costly vehicle prices, it is rare for young people to own their own vehicles. Under these specific circumstances that are present in South Korea, people who can drive are more likely to be of an older age and financially capable of purchasing and maintaining their own vehicles. According to a study that examined the relationship between political orientation and attitudes and behaviour with respect to the H1N1 virus crisis of 2009, people who preferred traditional news sources were less concerned about the flu outbreak. Considering these facts, we can conclude that radio listeners in South Korea are more likely to be more conservative and thus are less likely to engage in preventive behaviours [38].

Furthermore, trust in the information found on social media has a positive impact on preventive behaviours. With technological advances, the information circulated via social media has become more tangible and interactive [39, 40]. Given that people can upload first-hand information while on the scene and share specific information demanded by the people, social media plays a crucial role during crisis events [41]. However, since social media does not have strict controls over circulated information, there is much misinformation and fake news found in the online space [42]. Thus, to draw the most from social media, it is important to correct misinformation and deliver easy and understandable messages to the public [13].

Interpersonal communication has suggested to be an effective tool to motivate behaviour change [43]. In our study, we found that the trust placed in the information received from family and friends tended to have a positive impact on the respondents’ preventive behaviours. Individuals who directly experienced a crisis might provide detailed information of the crisis events to other individuals; this can prove helpful for others who are working to define the ongoing situation [44, 45]. Because of direct contact, interpersonal channels have been found to be more successful in engendering change [43].

In addition, our study revealed that if people trust government information more, then they will practice preventive behaviours more often. During these times of uncertainty, people are eager to find scientific and valid information about the pandemic. At this point, the government needs to provide reliable sources and recommendations to the public, and the public needs to trust the government’s capacity and follow their suggestions [46]. A relationship of trust between the government and the public play a key role in ending such a pandemic [47, 48]. Prior to COVID-19, South Korea experienced infectious epidemics such as influenza, A/H1N1, and the Middle East respiratory syndrome corona virus (MERS-CoV). These experiences have helped the Korean government learn the importance of having an epidemic response mechanism. To combat COVID-19, the Korea Centers for Disease Control & Prevention (KCDC) not only posted the national counts of the daily COVID-19 cases on its website but also offered daily infographics and charts, which were accessible via internet search engines and social media [2]. Through these channels, the public was able to easily access information about the pandemic, which might have positively influenced their adherence to preventive behaviours.

Our study does have several drawbacks. First, the data were collected based on the respondents’ self-reports. This could mean that the responses given might be different from information obtained through observation. Second, the cross-sectional survey could not measure the respondents’ changes over time. Given that the public’s response could have been different depending on the specific situation of the pandemic, a longitudinal study is required to examine the changes taking place over time. Third, the use of online panels limited the participation of certain important population groups. People who did not have access to the internet or social media were not able to participate in the survey. Therefore, the results might be applicable only to a certain group of users with online literacy.

Despite these limitations, an apparent strength of this study is that we explored people’s adherence to personal preventive behaviours towards COVID-19 and investigated the factors associated with preventive behaviours based on the HBM. Considering that the HBM has often been used in studies to explain people’s risk perception and health behaviour, it is anticipated that our study can explain preventive behaviours with respect to the sociocultural perspective by using various cues to take action variables, such as reliable communication channels and sources. Based on this aspect, this study suggests a relative community strategy that reflects the social and cultural backgrounds of South Koreans.

## Conclusion

Our study identified the factors associated with personal preventive behaviours found in South Korea. The factors of perceived susceptibility, self-efficacy, trust in the information from the radio, trust in the information on social networks, trust in the government, and trust in the information obtained from family and friends were significantly associated with preventive behaviours in the current study. Many people are still suffering from the consequences of the COVID-19 pandemic. To triumph against these consequences, it is important that we have precise risk perceptions and adhere to preventive behaviours that stem from credible information. Our study suggests that the government and health professionals should provide scientific information via a number of trusted media channels and sources and promote public health campaigns to help the public understand the concerned information and encourage them to engage in preventive behaviours.

## Supplementary Information

**Additional file 1.**

## Data Availability

The datasets generated during and analysed during the current study are not publicly available due to the necessity to protect data that are still under analysis for further studies; however, they are available from the corresponding author upon reasonable request.

## Abbreviations

HBM: Health Belief Model
WHO: World Health Organization
