# Supplementary material for: Determinants of adherence to personal preventive behaviours based on the health belief model: a cross-sectional study in South Korea during the initial stage of the COVID-19 pandemic
Source: BMC Public Health. 2022 May 11;22:944. doi: 10.1186/s12889-022-13355-x (PMC9092036; doi:10.1186/s12889-022-13355-x)
Supplement: Supplementary file 1 — Additional file 1. [file 12889_2022_13355_MOESM1_ESM.pdf]

Supplementary file (questionnaire)

I. Sociodemographic questions

SQ1. Sex

1. Male 2. Female

SQ2. Age

1. 20-29 2. 30-39 3. 40-49 4. 50-59

SQ3. Education

1. Less than bachelor's degree 2. Bachelor's degree 3. Master's or higher

SQ4. monthly household income (10,000 KRW)*

1. <300 2. 300 <600 3. ≥600

*KRW: Currency of Korea (1,000 KRW is approximately 1 USD)

SQ5 Marital status

1. Marital 2. never married 3. Divorced 4. Widowed 5. separated

II. Health Belief model

다음에 제시되는 **코로나 19 예방수칙과 관련하여 귀하께서 동의하는 정도**를 각각 답해 주세요.

Please answer the degree to which you agree with the following COVID-19 prevention measures.

No.	Questions	Scale (1=strongly disagree, 7=strongly agree)						
1	예방수칙을 수행하지 않으면 코로나 19에 감염될 가능성이 높아질 것 같다<br>If I do not take precautions, I think I will be more likely to	①	②	③	④	⑤	⑥	⑦

	be infected with COVID-19.							
2	예방수칙을 수행하지 않으면 코로나 19에 걸릴까봐 걱정된다.<br>If I do not take precautions, I worry that I will be infected with COVID-19.	①	②	③	④	⑤	⑥	⑦
3	만약 코로나 19에 감염된다면, 경제상황이 악화될 것 같다.<br>If I am infected with COVID-19, my financial status will likely become worse.	①	②	③	④	⑤	⑥	⑦
4	나는 코로나 19에 감염된다면, 오랫동안 힘들 것 같다.<br>If I am infected with the COVID-19, it will impact me severely.	①	②	③	④	⑤	⑥	⑦
5	예방수칙을 수행하면 안심이 된다.<br>I feel relieved when I follow the precautions.	①	②	③	④	⑤	⑥	⑦
6	예방수칙을 수행하면 코로나 19가 감염되는 것을 방지할 수 있을 것 같다.<br>If I follow the preventive behaviours, doing so will reduce the risk of getting infected with COVID-19.	①	②	③	④	⑤	⑥	⑦
7	예방수칙을 수행하는 일은 성가시고 불편한 일이다.<br>It is annoying and uncomfortable to follow the preventive behaviours.	①	②	③	④	⑤	⑥	⑦
8	예방수칙을 수행하면 사회적 관계가 멀어질 것 같다.<br>If I take precautions, I am afraid I will be socially disconnected.	①	②	③	④	⑤	⑥	⑦
9	나는 예방수칙을 잘 수행할 수 있다.<br>I am able to follow the preventive behaviours.	①	②	③	④	⑤	⑥	⑦

III. Health Behaviour

귀하는 코로나 바이러스 19가 발생한 이후, 다음과 같은 예방수칙을 실천하였습니까?

Have you adhered to the following preventive behaviours since the outbreak of COVID-19? (1=strongly disagree, 7=strongly agree)

No.	Preventive behaviours	Scale (1=strongly disagree, 7=strongly agree)						
1	Frequent hand hygiene	①	②	③	④	⑤	⑥	⑦
2	Respiratory etiquette	①	②	③	④	⑤	⑥	⑦
3	Wearing a mask	①	②	③	④	⑤	⑥	⑦
4	Environmental cleaning at home	①	②	③	④	⑤	⑥	⑦
5	Self-quarantine	①	②	③	④	⑤	⑥	⑦

IV. Cues to take action

귀하께서는 2020년 1월 한국에서 코로나바이러스 19가 발생한 이후부터 **다음의 각 정보원을 통해 공유된 "코로나 바이러스 19" 관련정보를 얼마나 많이 신뢰하십니까**?

How much do you trust the COVID-19-related information that you obtain through each of the following sources since the outbreak of COVID-19 in Korea in January 2020

No.	Reliable channels/resources	Scale (1=No trust at all, 7=most information can be trusted)						
1	Printed media	①	②	③	④	⑤	⑥	⑦
2	TV	①	②	③	④	⑤	⑥	⑦
3	Radio	①	②	③	④	⑤	⑥	⑦
4	Health care providers	①	②	③	④	⑤	⑥	⑦
5	Official government website	①	②	③	④	⑤	⑥	⑦
6	Social networks	①	②	③	④	⑤	⑥	⑦
7	Family and friends	①	②	③	④	⑤	⑥	⑦
